# Why do Ukrainian refugee women remain in a politically ambivalent host country? The case of Hungary

**DOI:** 10.3389/fsoc.2026.1922051

**Published:** 2026-09-16

**Authors:** Éva Berde, Andrea Csapó-Horváth, Sándor Remsei

**Affiliations:** 1 Department of International and Applied Economics, Kautz Gyula Faculty of Business and Economics, Széchenyi István University, Győr, Hungary; 2 Department of International Studies and Communication, Faculty of Education, Humanities and Social Sciences, Széchenyi István University, Győr, Hungary

**Keywords:** continued residence, everyday experience, Hungary, political communication, temporary protection, Ukrainian refugee women

## Abstract

**Introduction:**

The prolonged displacement of Ukrainians after the full-scale Russian invasion has raised questions about why refugees remain in particular host countries even when the broader political environment is not consistently welcoming. Hungary provides a distinctive case in which increasingly exclusionary national political communication coexisted with legal protection, local assistance, and relatively stable everyday conditions. The aim of this study is to understand how Ukrainian refugee women made sense of their continued residence in Hungary and how they interpreted the relationship between national political communication and their everyday experiences.

**Methods:**

The study uses a qualitative longitudinal design based on two rounds of interviews. The first round, conducted in late 2024 and early 2025, included eleven Ukrainian refugee women who had already lived in Hungary for at least two and a half years. The second round was conducted in December 2025. Six participants were interviewed directly, while factual status information was available for the remaining five.

**Results:**

The analysis showed that continued residence developed gradually rather than resulting from a single decision to settle permanently. Participants’ accounts highlighted employment, pre-existing family and labor migration connections, geographical proximity to Ukraine, children’s schooling, and the organization of everyday life as circumstances associated with increasing stability. At the same time, pessimistic expectations concerning security, reconstruction, and future living conditions in Ukraine made return appear unrealistic in the near term. Follow-up information showed that ten of the eleven participants remained in Hungary, while the six women interviewed directly expressed little immediate intention either to move onward to another country or to return to Ukraine. These women were aware of increasingly hostile political messages concerning Ukraine, yet such rhetoric was not generally reflected in their established interpersonal relations and everyday experiences. Temporary protection under EU rules also helped preserve lawful residence and access to employment and essential services.

**Discussion:**

The findings indicate that understanding refugees’ continued residence requires attention to how they perceive and navigate everyday living conditions, household circumstances, legal security, and available alternatives, alongside national political communication.

## 1 Introduction

The full-scale invasion of Ukraine by Russia in February 2022 triggered one of the largest refugee movements in Europe since the Second World War. Within a short period, millions of Ukrainians fled their homes and sought protection in other countries. By the end of 2025, approximately 5.9 million refugees from Ukraine were living outside the country, most of them in Europe (Porteous, 2026). Neighboring countries were important destinations for refugees from Ukraine. At the end of 2025, approximately 972,300 refugees from Ukraine were recorded in Poland and 138,800 in Slovakia, corresponding to about 26.6 and 25.7 refugees per 1,000 inhabitants, respectively. Germany, although not directly neighboring Ukraine, was the largest host country worldwide for refugees from Ukraine at the end of 2025, with approximately 1,205,700 refugees, corresponding to about 14.4 per 1,000 inhabitants. In Hungary, which also shares a border with Ukraine, approximately 64,800 refugees from Ukraine were recorded, corresponding to about 6.8 per 1,000 inhabitants (Eurostat, 2026; UNHCR, 2026). Thus, despite its geographical proximity to Ukraine, Hungary hosted substantially fewer refugees from Ukraine, both in absolute terms and relative to population, than Poland or Slovakia. Hungary is therefore relevant to our analysis not because of the scale of refugee reception, but because it provides a distinctive setting in which continued refugee residence coexisted with increasingly exclusionary national political communication.

Against this background, it is relevant to ask why some displaced Ukrainians continued to reside in Hungary several years after the initial phase of displacement. The Hungarian setting combines geographical proximity to Ukraine and pre-existing cross-border labor and family networks with a political environment that has often been characterized by restrictive migration discourse and highly politicized debates about migration (Kyriazi, 2022; Vidra and Messing, 2025). These circumstances make Hungary useful for examining how practical conditions that may facilitate continued residence interact with a less welcoming national political environment.

This tension became more pronounced as public communication about Ukraine grew increasingly critical and politically polarized (Feischmidt et al., 2025; Sik and Krekó, 2025; Takácsy, 2025; Vidra and Messing, 2025). Before the Hungarian parliamentary election of April 12, 2026, anti-Ukrainian and anti-Zelenskyy messages were highly visible in government communication, including large-scale billboard campaigns (Zsiros, 2025). The political context examined in this study refers primarily to this period. Following the election and the formation of a new government, these billboards were removed and official rhetoric toward Ukrainians changed substantially. The experiences analyzed here, however, were formed during the preceding period. In this article, we use the term politically ambivalent to refer to the coexistence of exclusionary national political communication with legal and practical conditions that nevertheless allowed Ukrainian refugees to establish and maintain everyday lives in Hungary. Accordingly, we ask: how do Ukrainian refugee women interpret the circumstances of their continued residence in Hungary when increasingly exclusionary national political communication coexists with relatively stable everyday conditions?

In this article, we address this question through a qualitative study of Ukrainian refugee women who had already been living in Hungary for a relatively long period. Although the literature on Ukrainian displacement expanded rapidly after 2022, many studies were conducted shortly after the full-scale invasion of Ukraine and therefore focused on relatively recent arrivals, while fewer have examined refugees’ experiences after a longer period of residence in the host country. By examining women who had remained in Hungary for several years and revisiting their circumstances over time, our study provides insight into the circumstances interviewees associated with their continued residence in a politically ambivalent host-country environment. We focus particularly on the coexistence of exclusionary national political communication and relatively stable everyday conditions, while limiting our conclusions to the experiences of the women interviewed.

Our study contributes to the literature in two main ways. Empirically, it adds evidence on the longer-term experiences of Ukrainian refugees in Hungary, a context that has received relatively limited attention in research on Ukrainian displacement. Conceptually, it highlights the importance of considering not only policy frameworks and an increasingly exclusionary national political discourse, but also the everyday social environments in which refugees live and work and the practical conditions that may support continued residence. The remainder of the paper reviews the relevant literature, outlines the research design, and presents the empirical findings. The Discussion summarizes the main findings, considers the study’s limitations, and outlines directions for future research.

## 2 Literature review and theoretical background

This section presents a focused narrative review combining foundational migration theory with recent empirical research on Ukrainian displacement in Europe. Earlier studies are included where they provide relevant concepts on migration decisions, settlement, and integration. The review centers on how continued residence can coexist with increasingly exclusionary national political communication. It therefore considers employment, migration networks, household strategies, children’s education, geographical proximity, and return or onward-migration intentions as factors that may help explain continued residence despite increasingly exclusionary national political communication.

### 2.1 The central puzzle: political discourse and continued residence

Migration decisions are rarely determined by formal policies or political discourse alone. Classical migration theories emphasize labor-market opportunities, migrant networks, and family strategies in shaping destination choice and subsequent migration trajectories (Castles et al., 2014; Massey et al., 1993). These factors may remain influential after arrival because social connections, employment opportunities, and household arrangements affect the feasibility of remaining, returning, or moving onward. More recent approaches conceptualize migration decision-making as an evolving relationship among aspirations, capabilities, and contextual constraints (Czaika et al., 2021). Refugees may wish to return, remain, or move elsewhere, but their capacity to realize these preferences depends on resources, family obligations, legal conditions, and perceived alternatives. A location initially regarded as temporary may therefore gradually become a place of longer-term residence as employment, social ties, and children’s educational arrangements develop (Crawley and Hagen-Zanker, 2019; Panzaru and Harding, 2025).

An additional perspective concerns the EU Temporary Protection Directive (Council directive 2001/55/EC, 2001), which was activated for people displaced from Ukraine in March 2022 through Council Implementing Decision (EU) 2022/382 (2022). The scheme granted eligible displaced persons immediate legal residence and access to employment and key services without requiring the usual individual asylum procedure. Its practical effects, however, depended on how these rights were implemented at national and local levels. Evidence from Poland shows that insufficient central administrative and financial support led municipalities, nongovernmental organizations, private actors, and supranational organizations to develop a partly fragmented support structure (Łukasiewicz et al., 2026). Local responses varied, with larger cities more likely to develop longer-term integration activities, although civil society and municipal cooperation remained important across different settings (Bielewska et al., 2025). Similar patterns were found in Italy, where weaknesses in the national reception system increased the role of municipalities and third-sector organizations (Bassoli and Campomori, 2026).

These findings show that relatively successful everyday inclusion does not necessarily require an active or consistently supportive central government. It may instead emerge when temporary protection limits formal exclusion and local institutions and civil society organizations translate legal rights into accessible services. In Hungary, Feischmidt et al. (2025) describe this process as “constrained localism”: civic and municipal alliances provided support as an alternative to the central state’s hostile migration policy, although these arrangements remained uneven and resource-dependent. This illustrates how local institutional support may partially diverge from national political discourse.

### 2.2 Interacting mechanisms shaping continued residence

Employment is one of the most frequently identified mechanisms supporting stability after displacement. Research in European host countries shows that Ukrainian refugees often enter the labor market relatively quickly, although many work below their formal qualifications (Aigner et al., 2025; Kosyakova et al., 2024). Occupational downgrading reflects language difficulties, non-recognition of qualifications, childcare responsibilities, and the need to earn income soon after arrival. Even employment below a person’s former professional level may provide financial independence and make continued residence more feasible. Its importance may also depend on household arrangements and pre-existing migration networks. A partner already working in the destination country may provide accommodation, employer contacts, information, and familiarity with local institutions, thereby reducing the uncertainty and practical costs of arrival and facilitating access to employment and other resources (Castles et al., 2014; Massey et al., 1993).

Remaining close to Ukraine can make it easier to maintain family relationships, obtain information, visit relatives, and preserve the possibility of return. Pre-existing labor migration and cross-border family connections are particularly relevant in Central and Eastern Europe, where mobility between Ukraine and neighboring countries existed before the full-scale invasion (Fedyuk and Kindler, 2016; Surányi and Bognár, 2025).

Children’s education may become increasingly important after arrival. Research on Ukrainian refugees shows that return intentions are shaped not only by individual preferences but also by household composition, partners’ circumstances, and children’s educational trajectories (Van Tubergen et al., 2024). As children learn the host-country language, form friendships, and progress through local schools, another move may become more disruptive and costly for the family. Schooling may therefore become a stronger reason to remain as residence lengthens. The concept of “active waiting” is also relevant. Rock and Yanaşmayan (2024) use the term to describe how displaced Ukrainian mothers create stability for their children while remaining uncertain about whether return will become possible. Waiting is therefore not passive but includes working, organizing schooling and childcare, maintaining transnational family relationships, and establishing manageable everyday routines. Other qualitative research similarly emphasizes the trauma experienced by displaced Ukrainian women and their resilience in rebuilding daily life in host societies (Baran et al., 2024; Preiss et al., 2024).

Continued residence is shaped not only by conditions in the host country but also by expectations concerning the country of origin. Return intentions depend on security, housing, employment prospects, household circumstances, and expectations regarding reconstruction (Ravenna et al., 2024; Van Tubergen et al., 2024). Many displaced Ukrainians express a desire to return eventually, while fewer consider return realistic in the near future. Continued warfare, damaged infrastructure, economic uncertainty, and concerns about children’s futures may make return impractical, allowing temporary arrangements in the host country to become more stable over time (Vakhitov et al., 2025).

Continued residence and return are not the only possible trajectories. Refugees may also consider onward migration when another country offers better employment opportunities, stronger networks, or more favorable institutional conditions. Research on secondary migration shows that trajectories may remain open after arrival and change in response to differences between destination countries and evolving household circumstances (Brekke and Brochmann, 2015).

### 2.3 Political reception and everyday experience

The reception of Ukrainian refugees in Europe was initially more favorable than that of several earlier refugee groups. Scholars have linked this to geographical and cultural proximity, geopolitical identification, and narratives portraying Ukrainians as particularly deserving of protection (Costello and Foster, 2022; Letki et al., 2025). The concept of “promising victimhood” captures how refugees may be seen both as legitimate victims and as people expected to work, adapt, and become accepted members of the host society (Mickelsson, 2025; Welfens, 2023). Such favorable reception may weaken as displacement becomes prolonged and host societies experience economic pressures or “refugee fatigue” (Baran et al., 2024). In Hungary, relatively favorable representations of Ukrainian refugees coexisted with increasingly critical political communication about Ukraine and its leadership (Takácsy, 2025; Vidra and Messing, 2025). The present study examines whether interviewees themselves distinguished between such political messages and their everyday experiences as refugees.

Refugees encounter the host country not only through national political discourse but also through workplaces, schools, administrative institutions, neighbors, churches, and volunteer organizations. These settings may reproduce exclusionary national messages but may also operate according to different institutional and interpersonal norms. Ager and Strang’s (2008) framework identifies employment, education, social connections, and rights as important dimensions of refugee integration and thus helps locate the domains in which inclusion or exclusion is experienced, although it does not directly explain why these experiences may diverge from national political discourse. This possible divergence is particularly relevant in Central and Eastern Europe, where proximity to Ukraine coexists with substantial differences in political and institutional responses.

### 2.4 Central European reception and the Hungarian case

The displacement of Ukrainians after 2022 made Central and Eastern European countries increasingly important host societies. Poland, Czechia, Slovakia, and Hungary share geographical proximity to Ukraine but differ in the scale of reception, institutional arrangements, labor-market opportunities, public attitudes, and political communication. Comparative research shows that these countries became important destinations rather than functioning only as transit locations (Pędziwiatr and Magdziarz, 2023), while other studies emphasize humanitarian assistance and support in Central European reception contexts (Seberini et al., 2025).

Countries initially perceived mainly as transit locations may become places of longer-term residence when refugees find employment, establish social relationships, and encounter manageable living conditions (Panzaru and Harding, 2025). Pre-existing labor migration and cross-border family networks may further facilitate this process. In Hungary, Ukrainian labor migration and cross-border family and employment relationships predated the full-scale invasion, so the country was not an entirely unfamiliar destination for some displaced Ukrainians (Surányi and Bognár, 2025).

At the same time, Hungary has been characterized by restrictive migration discourse and highly politicized debates about migration (Kyriazi, 2022). Ukrainian refugees were initially portrayed relatively favorably in 2022, although this occurred within a strongly government-shaped media environment (Vidra and Messing, 2025). Political communication later became increasingly critical of Ukraine and its leadership, presenting Ukraine as an economic, political, or security threat and portraying Ukrainian and European political leaders as acting against Hungarian interests (Takácsy, 2025). These campaigns fit a broader pattern of recurrent moral panics and the construction of symbolic enemies (Sik and Krekó, 2025), while billboard campaigns have long served as instruments of political contention and message control in Hungary (Nagy, 2016), although the anti-Ukrainian billboard campaign was discontinued after the 2026 parliamentary election. Yet national political communication was only one component of refugee reception: civic organizations, municipalities, churches, volunteers, schools, and employers continued to provide practical assistance (Feischmidt et al., 2025). The Hungarian environment can therefore be described as politically ambivalent, with exclusionary national communication coexisting with legal protection, local assistance, and relatively stable everyday institutions.

The literature discussed above has examined Ukrainian refugees’ reception and integration over time, including labor-market opportunities, return intentions, public attitudes, media representation, political communication, and humanitarian support. Much less is known, however, about how Ukrainian refugee women themselves interpret the relationship between national political messages and their everyday experiences after several years of residence. Our study addresses this gap by examining how everyday experiences relate to continued residence in Hungary despite hostile political communication.

## 3 Materials and methods

### 3.1 Research design, participant recruitment, and sample

The study uses a qualitative longitudinal design to explore how Ukrainian refugee women made sense of their continued residence in Hungary during a period characterized by increasingly exclusionary national political communication. More specifically, the study explores how participants described the circumstances of their continued residence and how they interpreted the relationship between national political communication and their everyday experiences in Hungary. The analysis follows an interpretative orientation in which participants’ accounts are treated as situated interpretations of their experiences rather than as direct evidence of objective causal relationships.

The empirical material consists of two rounds of interviews. The first round was conducted at the end of 2024 and the beginning of 2025, when the participants had already been living in Hungary for at least two and a half years after the full-scale invasion in February 2022. The follow-up took place in December 2025. The individual refugee woman constitutes the unit of analysis because the study is based on women’s personal experiences and interpretations. Participants frequently described household-level circumstances involving partners’ employment, children’s schooling, and cross-border family relations. These circumstances are analyzed as they were perceived and described by the individual participants, rather than as a separate household-level unit of analysis.

The first round included eleven Ukrainian refugee women residing in Hungary, all of whom had received temporary protection under EU rules. Eligible participants were Ukrainian refugee women who had already lived in Hungary for at least two and a half years and who agreed to participate in the interview study. Recruitment relied on two existing sources of contact because access to this population was limited. One source was a Hungarian volunteer support group assisting Ukrainian refugees. Members of the group asked women who had turned to them for assistance whether they would be willing to be contacted about the research and, if so, whether their telephone number could be shared with the researchers. Some women who initially expressed willingness to participate were not eligible because they had lived in Hungary for less than two and a half years. Others initially agreed to be contacted but did not respond when approached by the researchers. Approximately twice as many women provided their telephone numbers as ultimately participated in the study. Participation in the research was entirely voluntary and was not a condition of receiving assistance from the group.

The second source consisted of participants in an earlier questionnaire survey of Ukrainian refugees conducted by two of the authors of the present study. In that survey, respondents had been asked whether they were willing to provide an email address for possible future research. Twelve women did so, of whom three eligible women subsequently agreed to participate in the present interview study. Recruitment through the two sources took place in parallel. Recruitment ended when no additional eligible women from these sources agreed to participate. Theoretical saturation was not used as a formal stopping criterion. However, recurring topics and experiences became apparent across the interviews, and the research team considered the resulting material sufficient to address the research question.

The participants differed in age, family circumstances, and professional background. Almost all had arrived with children, and six had partners who had already been working in Hungary before the full-scale invasion. For comparison, a 2024 UNHCR socio-economic survey of 795 refugee households in Hungary, representing 1,824 people who had fled Ukraine, found that 63% of those assessed were women and girls. Among the active working-age population aged 18–59, 73% were female, and close to 40% of surveyed households consisted of a single woman with children (UNHCR, 2024). However, this source does not provide information on the prevalence of pre-existing labor-market or family connections to Hungary before the full-scale invasion, and we did not identify other available sources providing such information. We therefore cannot assess how typical the high proportion of such prior connections in our sample was within the broader Ukrainian refugee population in Hungary. The sample was purposive and network-based rather than statistically representative. Because recruitment relied partly on a volunteer support network, some participants may have had access to forms of assistance and social support that were not equally available to other Ukrainian refugees in Hungary. This limits the extent to which the findings can be generalized to Ukrainian refugees in Hungary more broadly. At the same time, the participants’ accounts provide insight into a particular pathway of longer-term residence in Hungary, including the role they attributed to pre-existing labor and family connections, practical support, social ties, and other everyday circumstances associated with their continued residence.

A particular strength of the study is its focus on women with a relatively long period of residence in Hungary. This makes it possible to examine experiences of longer-term residence rather than only the initial phase of displacement, which has been the focus of much of the earlier research on Ukrainian refugees. At the same time, the findings primarily reflect the experiences of women who remained in Hungary and offer very limited evidence on refugees who moved onward to another country.

### 3.2 Interview procedures

The first round consisted of semi-structured in-depth interviews, all conducted by the same author. These conversations were carried out either face to face or through the Viber application, depending on participants’ circumstances. A common interview guide ensured that the same broad topics were covered with all participants while allowing the interviewer to ask follow-up questions and explore issues raised by individual respondents. The discussions addressed the circumstances of leaving Ukraine and arriving in Hungary, employment, family arrangements, social relations, experiences in the host country, and expectations regarding the future in both Hungary and Ukraine.

Each first-round interview lasted approximately 60–90 min and was conducted in Russian. Although Ukrainian was the primary language for most participants, Russian was also familiar to them, as they had spoken it since childhood. Some participants noted that Ukrainian was preferred exclusively in certain refugee groups. They themselves, however, distinguished between Russian military aggression and the Russian language, as well as between the aggressors and Russian-speaking people they had known in everyday life. The use of Russian therefore did not appear to create communication difficulties or emotional discomfort during the interviews. Participation was voluntary, and all participants provided informed consent after receiving information about the aims of the research and the handling of their data. To protect anonymity, participants were not asked to sign written consent forms, and their names and identifying details are not reported in the article. All first-round interviews were audio-recorded with participants’ consent and subsequently transcribed verbatim. These transcripts formed the main qualitative material for the analysis.

The second round consisted of shorter semi-structured follow-up interviews conducted by the same author in December 2025 through Viber or by telephone. Six of the original participants were interviewed directly, information on four others was obtained indirectly through peers who knew them, and one woman had moved to Belgium. The follow-up focused on changes since the first interview, participants’ current living conditions, experiences of hostile or exclusionary behavior, and their plans and expectations regarding the future. Notes were taken during the direct follow-up conversations, and detailed descriptions were prepared afterward. Because peer-reported information is more appropriate for establishing factual circumstances than for examining subjective experiences, indirect information was used only for factual or status-related purposes, such as determining whether a participant was still living in Hungary. Interpretive claims concerning experiences of political hostility, everyday social relations, and future intentions are therefore based only on the six direct follow-up interviews. This distinction is particularly important because the study focuses on participants’ own interpretations and perceptions.

### 3.3 Data analysis

The interview material was analyzed using inductive thematic content analysis. The author who conducted the interviews also carried out the initial coding of the first-round interview transcripts. In the first stage, each transcript was read line by line, and important statements, topics, and meaning units were identified and assigned short descriptive codes. These initial codes remained close to the participants’ own words and accounts. In the second stage, related codes were grouped into broader categories, after which the main themes were identified. The coding process was iterative: transcripts were reread several times, and codes and categories were progressively refined until a final coding structure was established. The initial coding was not based on a predefined set of explanatory factors. Categories and themes were developed from recurring patterns in the participants’ accounts. The same analytical principles were applied consistently across the first-round transcripts and the notes from the direct follow-up interviews. The procedure followed an inductive thematic-analysis consistent with Naeem et al. (2023).

Five broad themes emerged from the initial analysis: origin and family; traumatic experiences; settlement and satisfaction; future prospects; and the future of Ukraine, including the possibility of return. After the themes had been checked against the interview material, they were finalized, and quotations were selected to illustrate the interpretations. The author who conducted and coded the interviews also translated the codes and coding framework into English, which enabled all authors to analyze and interpret the material jointly. For the present analysis, the coded material was subsequently re-examined in relation to the study’s central research question, comparing experiences across participants and paying particular attention to the relationship between national political communication and everyday experience. The coded material was then compared across participants to examine how the themes that emerged from their accounts related to one another and to their continued residence in Hungary.

The follow-up material was analyzed alongside the first-round interviews to identify continuity and change over time. Particular attention was also given to cases that did not fit the dominant pattern, including accounts of dissatisfaction, exclusion, or discrimination. These cases were considered analytically rather than treated as isolated anecdotes, allowing the study to examine both the observed separation between political rhetoric and everyday experience and circumstances in which this separation was less evident. The use of the same interviewer in both rounds provided continuity in the interviewer-participant relationship and facilitated discussion of changes since the first interview. At the same time, continuity may also have influenced how participants presented their experiences. All authors are based in Hungary, and the politically sensitive nature of some of the questions requires attention to researcher positionality. Participants may have differed in their willingness to discuss political hostility, discrimination, or dissatisfaction with life in Hungary when speaking to a researcher working in the host country. The limited reporting of hostility is therefore interpreted cautiously and is not taken as evidence that discrimination or exclusion was absent either from participants’ lives or from the experiences of Ukrainian refugees in Hungary more generally.

## 4 Results

The results are organized around the participants’ pathways into Hungary, the circumstances they associated with continued residence, their expectations regarding return or onward migration, and the relationship between political hostility and everyday experience.

### 4.1 Arrival pathways and the transition from temporary refuge to residence

The participants arrived in Hungary under different circumstances, although all had been displaced by the full-scale invasion of Ukraine. For several women, departure was sudden and followed experiences of heavy bombardment or military occupation. Some had spent days or weeks in shelters or basements before leaving Ukraine, often traveling with their children. One participant from the Kherson region recalled: “For nine days we stayed in the basement. We could hear explosions all the time and did not know if the house would still be standing the next morning.” These experiences left little opportunity to prepare for migration or systematically compare possible destination countries.

The interviews nevertheless revealed two somewhat different pathways into Hungary. For six of the eleven women, Hungary was not an entirely unfamiliar destination: their partners had already been working there before the full-scale invasion. Their arrival therefore took place within an existing cross-border labor and family arrangement. The presence of a partner already living and working in Hungary gave these women an immediate practical connection to the country and may have reduced some of the uncertainty associated with displacement. For the other participants, the choice of Hungary reflected a combination of chance, geographical proximity to Ukraine, and recommendations from Ukrainians already living in the country. Thus, although all participants arrived as refugees from the war, their starting conditions differed substantially: for more than half of the sample, wartime displacement was superimposed on an already existing migration connection to Hungary. For most participants, however, the initial expectation was that their stay in Hungary would be temporary. One woman who arrived in Budapest with her children expected to stay only a few weeks until conditions in Ukraine stabilized. As the war continued and return became increasingly uncertain, however, temporary arrangements gradually developed into longer-term residence. This transition was not the result of a single decision made upon arrival. Rather, it developed over time as participants found ways to organize employment, family life, schooling, and other aspects of everyday life in Hungary.

Participants also described their initial experiences in Hungary as relevant to this process. Several interviewees received assistance from volunteer organizations, churches, or humanitarian groups, including temporary accommodation, food, and help with administrative procedures. Encounters with state authorities during mandatory registration were also generally described positively. These experiences did not eliminate later difficulties, particularly those related to language and employment, but participants generally described them as contributing to an initial sense that everyday life in Hungary was manageable.

### 4.2 Circumstances associated with continued residence

Employment was an important element of the stability participants gradually established in Hungary. Of the eleven women, nine wished to work and all nine had found employment, while the remaining two were not seeking paid work at the time of the interviews and relied on their partners’ income to support the family. With one exception, however, the jobs were below the women’s previous level of qualification. One participant explained: “In Ukraine I had a professional job, but here I work as a cleaner. It is not what I studied for, but at least I can earn money and support my family.” Thus, occupational downgrading was common, but employment nevertheless provided income and reduced dependence on external assistance. One woman was an exception to the general pattern of occupational downgrading. She was able to work in the legal field, which corresponded to her original education. She had also acquired a relatively good command of Hungarian. However, she still could not work in this field formally because her qualification had not yet been recognized in Hungary. She was therefore working unofficially while studying to obtain a qualification recognized in Hungary.

Employment was also important for the four women in paid work whose partners were already employed in Hungary. At the same time, these households benefited from the relative financial security provided by the partner’s income, which gave the women greater flexibility to seek employment that was compatible with their children’s needs and other family responsibilities. Geographical proximity to Ukraine was also described as relevant to the stability they had established in Hungary. Participants valued the possibility of maintaining contact with relatives and of reaching Ukraine relatively easily if circumstances required it. One interviewee explained: “Hungary is close. If something happens at home, we can still go back or our relatives can come here.” Proximity was therefore described as making it easier to maintain cross-border family relationships while establishing everyday life in Hungary.

Based on the interview accounts, language emerged as a substantial difficulty rather than as a reason for continued residence. The woman mentioned previously had acquired a relatively good command of Hungarian, but almost all other participants described Hungarian as difficult to learn. Limited proficiency restricted employment opportunities and complicated everyday communication. Work schedules, childcare responsibilities, and long working hours also made regular participation in language courses difficult for some women. Nevertheless, these difficulties had not led them to leave Hungary. Instead, participants generally described learning Hungarian as a continuing challenge to be managed within the lives they had already established. Children’s education, by contrast, became increasingly important in participants’ accounts of continued residence. As children learned Hungarian, formed friendships, and progressed through school, another move could become increasingly disruptive for the household. One mother described this dilemma directly: “My son already speaks Hungarian and has friends here. If we moved on or returned to Ukraine now, he would have to start everything again.” Younger children were generally described as adapting more easily to Hungarian-language schooling, whereas older children sometimes experienced greater difficulties and, in several cases, continued part of their education in Ukrainian, either locally or online.

### 4.3 Return expectations and the narrowing of alternatives

The first-round interviews included a direct question about participants’ expectations for the future and their assessment of developments in Ukraine. Not all participants discussed this issue in detail, but those who did generally expressed pessimistic expectations regarding the possibility of return in the near future. Several referred to the destruction of infrastructure, the widespread presence of mines, and the economic consequences of the war as obstacles that would remain even if the fighting ended. Some also mentioned corruption and political uncertainty as factors that could slow reconstruction and stabilization. One participant reflected more broadly on the conflict by saying that politics had turned “two brotherly nations against each other.” Among those who discussed return in detail, permanent return was generally not regarded as realistic in the foreseeable future. Only one woman said that she would return as soon as possible, although she also considered such a return unrealistic under the prevailing circumstances. These expectations were closely connected with the stability participants had gradually established in Hungary, while conditions in Ukraine made return appear increasingly uncertain. Continued residence therefore did not necessarily mean that participants had abandoned the possibility of returning permanently. Rather, for many of those who discussed the issue, return remained a possible future option whose timing had become increasingly difficult to envisage. Visiting Ukraine if security conditions improved was considered more realistic than permanent return in the near future.

As noted above, follow-up information showed that ten of the original eleven participants were still living in Hungary. Interpretive conclusions about future plans are based only on the six women interviewed directly. Among these women, there was little indication of an immediate intention either to return to Ukraine or to move onward to another country. Their accounts suggested that the alternatives to remaining in Hungary had not become more attractive since the first interviews. At the same time, they did not necessarily describe their residence in Hungary as permanent or irreversible. Rather, continued residence remained linked to the practical stability they had established and to uncertainty about future conditions elsewhere.

### 4.4 Political hostility and everyday experience

The six women interviewed directly in the follow-up clearly perceived the increasingly critical political communication about Ukraine that characterized the period before the 2026 parliamentary election. Several referred specifically to the billboard campaigns and interpreted them as hostile toward Ukraine and Ukrainians. At the same time, they generally distinguished these political messages from their own everyday experiences in Hungary. They reported that previously established relationships with Hungarians had largely remained unchanged and that voluntary and church organizations continued to provide support. They also referred to the temporary protection granted under EU rules, which ensured access to basic rights and services, including employment, children’s education, and health care. However, some participants also expressed the view that Hungary should show greater solidarity with people fighting in Ukraine, in line with other European Union countries. They also indicated that if political hostility were to develop into a more direct threat to Ukrainians living in Hungary, onward migration to another European country could become a realistic option.

This separation between political communication and everyday experience was not complete. One participant reported that her child was not invited to the birthday party of her best Hungarian friend, although the two children remained on good terms and the child had previously visited her friend’s home several times. According to the participant, the friend’s parents had subsequently begun avoiding contact when the families met at school. In contrast, another participant described a teacher who made a particular effort to ensure that her son understood what was being taught and regularly praised his diligence and perseverance in front of the class. Experiences in the labor market were similarly mixed. One woman reported being told during a job interview that only Hungarian citizens were employed. Another encountered Ukrainian-language job advertisements and job fairs specifically targeting Ukrainian workers. However, some of the available jobs were relatively low-paid and monotonous.

These accounts show that experiences of inclusion and exclusion could coexist across different areas of everyday life. Hostile national political communication was not generally reflected in hostile interpersonal relations, although participants were not entirely free from exclusion or discrimination. The contrast between the supportive teacher and the exclusion experienced by another participant’s child, as well as between citizenship-based rejection and Ukrainian-language recruitment in the labor market, illustrates the uneven character of everyday reception. None of the six women interviewed directly in the follow-up reported that these negative experiences had led her to reconsider continued residence in Hungary.

## 5 Discussion, limitations, and future research

The findings indicate that Ukrainian refugee women’s longer-term stay in Hungary emerged gradually as their practical and family circumstances became more settled. Although Hungary was initially regarded by most participants as a temporary place of refuge, the passage of time altered the range of realistic options available to them. This was true not only for those whose partners were already working in Hungary, but also for the other participants. This interpretation is consistent with approaches that understand migration decisions as changing relationships among aspirations, capabilities, and contextual constraints (Czaika et al., 2021). It also supports research showing that an initially provisional destination may acquire greater importance as migrants establish routines, relationships, and responsibilities there (Crawley and Hagen-Zanker, 2019; Panzaru and Harding, 2025). The Hungarian case therefore illustrates a trajectory in which remaining did not necessarily imply a firm intention to settle permanently. Rather, the circumstances favoring departure became less compelling relative to the life participants had established in the host country.

Participants’ accounts indicate that pre-existing connections to Hungary were associated with different starting points in this process. For six women, a partner had already been employed in Hungary before the full-scale invasion, meaning that wartime displacement intersected with an earlier pattern of cross-border labor mobility. For the others, chance also played a role, alongside proximity to Ukraine and information from Ukrainians already living in Hungary. This distinction reinforces the importance attached by migration theory to household strategies and migrant networks (Castles et al., 2014; Massey et al., 1993). The interviews show that the presence of an employed partner did not make the women’s own labor-market participation unimportant, while employment was particularly important for those without a partner already working in Hungary. Most employed participants nevertheless worked below their previous qualification level, a pattern also documented among Ukrainian refugees elsewhere in Europe (Aigner et al., 2025; Kosyakova et al., 2024). The interviews therefore show that the women associated employment with greater stability, while also describing the constraints that accompanied labor-market entry. The participants’ high level of economic activity and their efforts to manage work, family responsibilities, and adaptation to everyday life also show some parallels with the idea of “promising victimhood” (Mickelsson, 2025; Welfens, 2023), according to which refugees may be regarded as deserving of protection while also being expected to work, adapt, and become self-supporting.

The interview material also highlighted the importance of family considerations and expectations about Ukraine for these trajectories. As children became more established in Hungarian schools and social environments, the costs of another relocation for the family could increase. This finding is consistent with Van Tubergen et al. (2024), who emphasize the importance of children’s educational circumstances in return intentions. It also relates to Rock and Yanaşmayan’s (2024) concept of “active waiting.” The women had not necessarily given up the idea of returning, but uncertainty about the future did not prevent them from organizing schooling, work, childcare, and other aspects of daily life in Hungary. In their accounts, Hungary’s location made it possible to maintain a sense of connection with Ukraine without making immediate return necessary. At the same time, the uncertain prospects they associated with postwar recovery weakened the attractiveness of return as a near-term option. In this way, proximity to Ukraine coexisted with an increasingly established everyday life in Hungary.

The clearest contribution of the study concerns the relationship between national political communication and participants’ own experiences. The women interviewed directly in the follow-up were aware of increasingly exclusionary messages concerning Ukraine before the 2026 parliamentary election, yet they generally did not experience a corresponding deterioration in their established day-to-day relationships. Local volunteer and church organizations continued to assist them, and previously established contacts with Hungarians largely persisted. The findings are therefore compatible with Feischmidt et al.’s (2025) notion of “constrained localism,” in which local and civic forms of support continue to operate within a less welcoming national political environment. Their temporary protection status was also important because it preserved lawful residence and access to employment, schooling, and health care despite the hostile tone of national political communication. In this sense, temporary protection functioned as a kind of institutional buffer between the state’s hostile rhetoric and the rights and services that nevertheless remained available to refugees in practice. More broadly, these findings suggest that refugees experience the host society through several levels at once and that national rhetoric, legal status, local institutions, and interpersonal relations need not move in the same direction.

The contrast was nevertheless not absolute. The exclusion of one participant’s child from a friend’s birthday celebration and the refusal to employ another participant because she was not a Hungarian citizen show that negative experiences were also present. At the same time, other accounts described supportive teachers and employers actively recruiting Ukrainian workers. The empirical picture is therefore one of uneven reception rather than either general hostility or general acceptance. The women also indicated that a more direct threat to Ukrainians living in Hungary could make moving elsewhere a realistic possibility. Political communication should consequently not be regarded as irrelevant simply because it had not yet outweighed the practical reasons for remaining.

Several limitations should be considered when interpreting these findings. Participant recruitment relied partly on existing social connections, and several women had received support through the same volunteer organization or through networks connected to it. This may have meant that some interviewees benefited from practical assistance and social support that were less accessible to other Ukrainian refugees in Hungary. In addition, six of the eleven participants had partners who were already employed in Hungary before the full-scale invasion. Such prior ties may have provided these women with practical advantages in finding accommodation, entering the labor market, obtaining support, and organizing family life. Consequently, the themes of employment, family networks, and increasing everyday stability may be especially visible in our sample, whereas the experiences of women who arrived without comparable connections to Hungary are represented to a lesser extent.

The study deliberately focuses on women who had already spent a relatively long period in Hungary. This is an advantage for examining experiences beyond the initial phase of displacement, but it means that the sample is dominated by women who stayed in Hungary and provides very limited evidence on those who moved onward to another host country. The study consequently cannot establish whether those who left Hungary encountered substantially different settlement conditions or experiences of political hostility. In addition, only six participants were interviewed directly in the second round, so interpretations of changing attitudes, interpersonal experiences, and future plans are restricted to those women. Finally, the same researcher conducted both rounds and performed the initial coding. This continuity facilitated longitudinal comparison but may also have affected participants’ presentation of politically sensitive experiences. Because all authors are based in Hungary, participants may not always have felt entirely comfortable reporting experiences of hostility or exclusion. Reports of limited hostility should therefore be interpreted cautiously and should not be taken to imply that discrimination was absent among Ukrainian refugees more generally.

These limitations also point to several directions for further research. Expanding the number of Ukrainian refugee women interviewed in Hungary could capture a wider range of experiences and settlement trajectories. It would also be valuable to include women who arrived in Hungary only after first spending time in another host country, as their assessments of Hungary may be shaped by comparative experience and by the circumstances that prompted onward migration. Another useful direction would be to examine whether the length of residence in Hungary influences women’s perceptions of the host country, including their views of everyday social relations, political communication, and future mobility options. Further follow-up research could also investigate whether the post-election change in hostile political communication altered refugees’ everyday experiences, perceived security, or willingness to remain in Hungary. Finally, comparing the views, experiences, and subsequent decisions of women who remained in Hungary with those of women who moved onward could help clarify how broader living conditions and settlement circumstances are associated with different migration trajectories.

## Data Availability

The data analyzed in this study are not publicly available and are not available upon request in order to protect the anonymity and confidentiality of the interview participants. Further inquiries can be directed to the corresponding author at mailto: berde.eva@sze.hu.
